# The Impact of Alpha-Syntrophin Deletion on the Changes in Tissue Structure and Extracellular Diffusion Associated with Cell Swelling under Physiological and Pathological Conditions

**DOI:** 10.1371/journal.pone.0068044

**Published:** 2013-07-05

**Authors:** Lesia Dmytrenko, Michal Cicanic, Miroslava Anderova, Ivan Vorisek, Ole Petter Ottersen, Eva Sykova, Lydia Vargova

**Affiliations:** 1 Institute of Experimental Medicine AS CR, v.v.i., Prague, Czech Republic; 2 Charles University, 2nd Faculty of Medicine, Prague, Czech Republic; 3 Center for Molecular Biology and Neuroscience and Department of Anatomy, University of Oslo, Oslo, Norway; Univ. Kentucky, United States of America

## Abstract

Aquaporin-4 (AQP4) is the primary cellular water channel in the brain and is abundantly expressed by astrocytes along the blood-brain barrier and brain-cerebrospinal fluid interfaces. Water transport via AQP4 contributes to the activity-dependent volume changes of the extracellular space (ECS), which affect extracellular solute concentrations and neuronal excitability. AQP4 is anchored by α-syntrophin (α-syn), the deletion of which leads to reduced AQP4 levels in perivascular and subpial membranes. We used the real-time iontophoretic method and/or diffusion-weighted magnetic resonance imaging to clarify the impact of α-syn deletion on astrocyte morphology and changes in extracellular diffusion associated with cell swelling *in vitro* and *in vivo*. In mice lacking α-syn, we found higher resting values of the apparent diffusion coefficient of water (ADC_W_) and the extracellular volume fraction (α). No significant differences in tortuosity (λ) or non-specific uptake (*k′*), were found between α-syn-negative (α-syn −/−) and α-syn-positive (α-syn +/+) mice. The deletion of α-syn resulted in a significantly smaller relative decrease in α observed during elevated K^+^ (10 mM) and severe hypotonic stress (−100 mOsmol/l), but not during mild hypotonic stress (−50 mOsmol/l). After the induction of terminal ischemia/anoxia, the final values of ADC_W_ as well as of the ECS volume fraction α indicate milder cell swelling in α-syn −/− in comparison with α-syn +/+ mice. Shortly after terminal ischemia/anoxia induction, the onset of a steep rise in the extracellular potassium concentration and an increase in λ was faster in α-syn −/− mice, but the final values did not differ between α-syn −/− and α-syn +/+ mice. This study reveals that water transport through AQP4 channels enhances and accelerates astrocyte swelling. The substantially altered ECS diffusion parameters will likely affect the movement of neuroactive substances and/or trophic factors, which in turn may modulate the extent of tissue damage and/or drug distribution.

## Introduction

Astrocyte swelling is an early hallmark of various pathological brain conditions, such as ischemia, hyponatremia and brain trauma, which are accompanied by transmembrane ionic shifts of Na^+^, Ca^2+^ and/or K^+^ and, as a consequence, water influx [1]. The aquaporin-4 (AQP4) channel, the predominant aquaporin in the central nervous system (CNS), has been proposed to modulate the function of nearby electrically excitable cells through the regulation of activity-dependent volume changes of the extracellular space (ECS) [2]. The activity/expression of aquaporins in the CNS is sensitive to different physiological as well as pathological stimuli. It was shown that in rodents, the expression of AQP4 protein in astrocytes is up-regulated in response to hyponatremia [3], after middle cerebral occlusion [4] and in cerebral edema caused by brain injury [5]. Conversely, down-regulation of the perivascular AQP4 pool was found after ischemia reperfusion [6]. In primary astrocytic cultures from mice lacking AQP4, it was demonstrated that the cells’ water permeability was reduced seven-fold as compared with wild type animals [7].

AQP4 is anchored by α-syntrophin (α-syn), an adapter protein in the dystrophin-associated complex [8]. Deleting α-syn results in the mislocalization of AQP4 and leads to a reduction in the amount of AQP4 in perivascular and subpial membranes, although the total AQP4 protein content in the brain remains unchanged [8]. Moreover, the AQP4 channel is co-expressed with Kir4.1, the inwardly rectifying K^+^ channel, which is believed to be involved in K^+^-spatial buffering. Thus, some studies have reported delayed extracellular K^+^ clearance in α-syn-negative or AQP4-negative mice [9]–[12]. Importantly, mice lacking AQP4 display reduced cerebral cytotoxic edema in response to water intoxication and stroke [13] as well as bacterial meningitis [14] with improved neurological deficit scores and better survival as compared with wild type animals. Furthermore, AQP4- or α-syn-negative animals show increased seizure threshold and severity [9], [11], [15], [16]. However, AQP4-deficient mice show greater brain water accumulation and a worse clinical outcome in models of vasogenic edema, such as brain tumor edema, brain abscess, cortical-freeze injury and obstructive hydrocephalus [17]–[19].

Cell swelling due to water redistribution accompanying physiological and pathological states leads to a compensatory shrinkage of the extracellular space volume, followed by changes in ECS composition and diffusion properties. The extracellular volume fraction (α) and tortuosity (λ) are the two ECS diffusion parameters that govern the diffusion of neuroactive substances through the volume of the ECS, the underlying mechanism of extrasynaptic (volume) transmission, which is not only an alternative mode of intercellular communication, but which can also modify synaptic transmission itself. Cell swelling-induced tissue remodeling with concomitant changes in the ECS diffusion parameters leads to the decreased ability of neuroactive substances to move within the ECS, thus impairing extrasynaptic transmission and may result in a functional brain deficit and/or damage of the nervous tissue [20], [21].

In the current study, we used the real-time iontophoretic tetramethylammonium (RTI-TMA) method [20] and complementary measurements of the apparent diffusion coefficient of water (ADC_W_) by diffusion-weighted magnetic resonance imaging (DW-MRI) to determine the impact of α-syn deletion and the resultant redistribution of AQP4 channels on the changes in tissue structure and extracellular diffusion associated with cell swelling under physiological as well as pathological conditions, both *in vitro* and *in vivo*.

## Materials and Methods

### Ethics Statement

All procedures involving the use of laboratory animals were performed in accordance with the European Communities Council Directive 24 November 1986 (86/609/EEC) and animal care guidelines approved by the Institute of Experimental Medicine, Academy of Sciences of the Czech Republic Animal Care Committee on April 17, 2009; approval number 149/2010. All efforts were made to minimize animal suffering and to reduce the number of animals used.

### Animals

Measurements were performed in cortical layers III–IV on both male and female 3–4-month-old mice homozygous for the targeted disruption of the gene encoding α-syntrophin (further identified as α-syn −/−). The α-syn −/− mouse line was originally generated by Stan Froehner and Marv Adams (Department of Physiology and Biophysics, University of Washington, Seattle, 98195, USA). Wild-type C57BL/6 mice were used as controls (further identified as α-syn +/+).

For *in vivo* experiments the animals were anesthetized with isoflurane (1.5% in a gas mixture of 35% O_2_/65% N_2_O) administered by a facemask, and their heads were fixed in a stereotaxic holder. Body temperature was maintained at 37°C by a heating pad. In experiments using the RTI method, the somatosensory cortex was partially exposed by a burr hole 1.5 mm caudal from bregma and 1.5 mm lateral from the midline, and the dura was carefully removed. The exposed brain tissue was bathed in warm (37°C) artificial cerebrospinal fluid (aCSF) containing in mM: 117 NaCl, 3 KCl, 35 NaHCO_3_, 1.25 Na_2_HPO_4_, 1.3 MgCl_2_, 1.5 CaCl_2_, 10 glucose, and 0.1 TMA^+^ (pH 7.4, 300 mOsmol/l).

For *in vitro* experiments, the mice were anesthetized by isofluran and decapitated. The brains were dissected out, and 400 µm thick coronal slices were prepared using a vibrating blade microtome (HM 400, Microm Int. GmbH). After cutting, the slices were maintained for 1–2 hours in aCSF. Recordings were performed at room temperature (22°C to 26°C) in a chamber perfused with a continuously bubbled (95% O_2_ and 5% CO_2_) aCSF at a flow rate of 4 mL/min.

### Experimental Models of Cell Swelling

The changes in cell volume associated with physiological or pathological states were simulated in the cortical slices by superfusion with either 10 mM K^+^ or hypotonic solutions (−50 mOsmol/l; H-50 and −100 mOsmol/l; H-100). Hypoosmotic solutions were prepared by reducing the NaCl content of the aCSF. The osmotic strength of the solutions was measured with a vapor pressure osmometer, thus “normal” aCSF had an osmolarity of 300 mOsmol/l, H-50 of 250 mOsmol/l and H-100 of 200 mOsmol/l. Solutions with an increased K^+^ concentration had a reciprocally reduced Na^+^ concentration.

Differences in cell swelling between α-syn +/+ and α-syn −/− mice during severe pathological states were examined in an experimental model of terminal ischemia/anoxia *in vivo*, evoked by cardiac arrest induced by the intraperitoneal administration of 1 ml saturated MgCl_2_. Electrocardiographic recording was used to monitor heart arrest.

### Immunohistochemistry

The 400 µm thick brain slices obtained prior to, after a 30 min incubation in H-50, H-100 or 10 mM K^+^ and after a 60 min washout were post-fixed in 4% paraformaldehyde in 0.1 M phosphate buffer for 3 hours, then placed stepwise into solutions with gradually increasing sucrose concentrations (10%, 20%, 30%) for cryoprotection. The coronal slices (40 µm thick) were prepared using a microtome (HM 400, Microm Int. GmbH, Waldorf, Germany). The slices were first incubated in blocking solution, which contained 5% Chemiblocker (Millipore, MA) and 0.2% Triton in 0.01 M phosphate buffered-saline. This blocking solution was also used as the diluent for the antisera. The slices were then incubated with the primary mouse antibody against glial fibrillary acidic protein (anti-GFAP coupled to Alexa 488;1∶800) at 4°C overnight (eBioscience, San Diego, CA, USA) and mounted using Vectashield mounting medium (Vector Laboratories, Burlingame, CA, USA). The tissue slices were then examined using an LSM 5 DUO spectral confocal microscope equipped with Arg/HeNe lasers and 40× or 63× oil objectives (Zeiss, Germany).

### Measurements of Extracellular Space Diffusion Parameters

**Figure 1:**
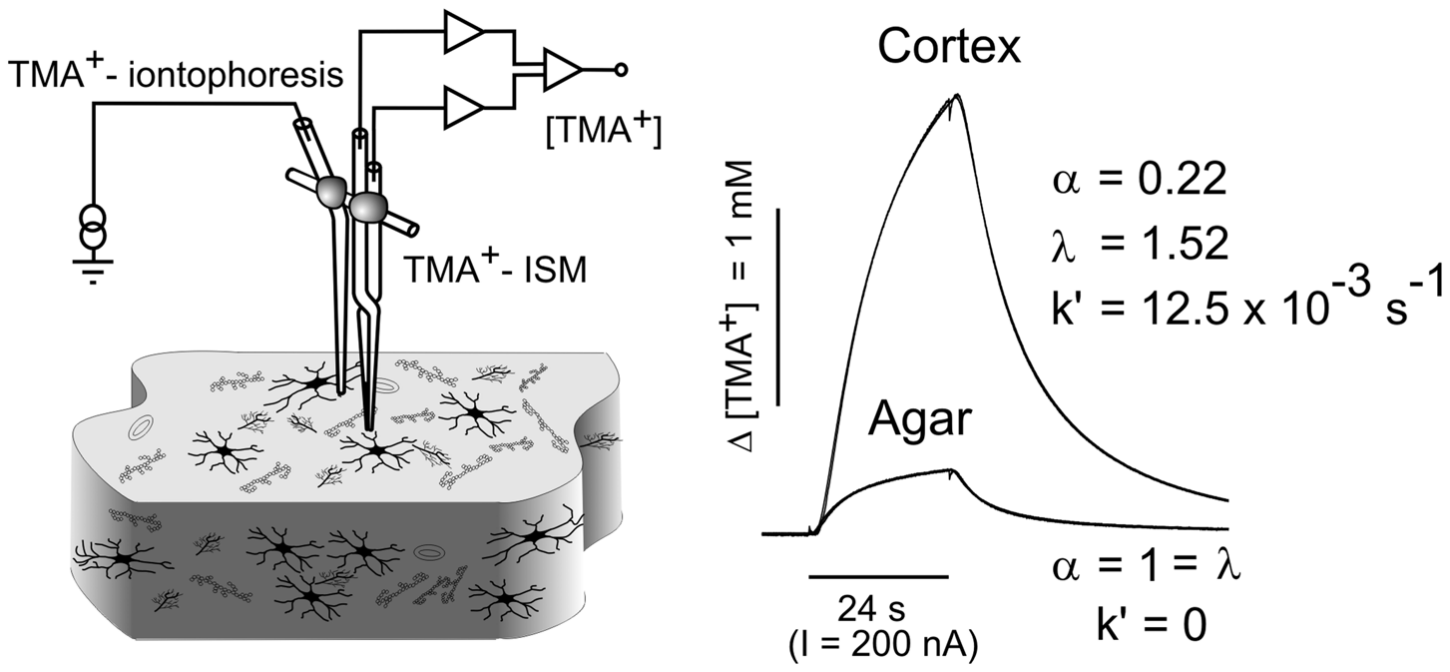
Experimental set-up and representative TMA^+^-diffusion curves in agar gel and healthy cortex. To stabilize the intertip distance of the electrode array, an iontophoretic micropipette and TMA^+^-selective microelectrode were glued together with dental cement (left). In the brain, where diffusion is constrained by various barriers and restricted to the extracellular space, the amplitude of the diffusion curve is much higher and its shape differs from the diffusion curve measured in agar gel, where by definition α = λ = 1 and *k′* = 0 (right).

The real-time iontophoretic method was used to determine the ECS diffusion parameters: ECS volume fraction α, which is the ratio of the volume of the ECS to total tissue volume in a representative volume of brain tissue (α = ECS/total tissue volume), tortuosity λ, which is defined as λ^2^ = *D/ADC* where *D* is the free diffusion coefficient and *ADC* is the apparent diffusion coefficient in the brain tissue, and non-specific concentration-dependent or independent uptake *k′* [22]. Briefly, an extracellular marker such as the tetramethylammonium ion (TMA^+^, Mw = 74.1 Da) that does not cross the cell membrane and is not toxic in small concentrations is administered into the extracellular space by iontophoresis (Fig. 1). The local concentration of TMA^+^ is measured with a TMA^+^-selective microelectrode (TMA^+^-ISM), and it is inversely proportional to the ECS volume. Double-barreled TMA^+^-ISMs were prepared by a procedure described in detail previously [23]. The tip of the ion-sensitive barrel was filled with a liquid ion exchanger for K^+^ (Corning 477317 or IE 190 from WPI) that is highly sensitive to TMA^+^ ions; the rest of the barrel was backfilled with 150 mM TMA^+^ chloride. The reference barrel contained 150 mM NaCl (Fig. 1). The electrodes were calibrated using the fixed-interference method before each experiment in a series of solutions of 150 mM NaCl +3 mM KCl with the addition of the following concentrations of TMA chloride (mM): 0.25, 0.5, 1, 2, 4, 8 and 16 mM.

An electrode array was made by gluing a TMA^+^-ISM to an iontophoretic micropipette with a tip separation of 100–200 µm (Fig. 1). Typical iontophoresis parameters were 20 nA bias current (continuously applied to maintain a constant transport number) and a +180 nA current step with a 24 sec duration to generate the diffusion curve. TMA^+^ diffusion curves were first recorded in 0.3% agar (Sigma-Aldrich, Steinheim, Germany) dissolved in a solution of 150 mM NaCl, 3 mM KCl and 1 mM TMACl, in which by definition α = 1, λ = 1 and *k′* = 0 (free-diffusion values). The diffusion curves obtained in agar were analyzed to yield the electrode transport number (*n*) and the free TMA^+^ diffusion coefficient (*D*) by curve fitting according to a modified diffusion equation using the VOLTORO program [22]. Diffusion curves were then generated in the somatosensory cortex at a depth of 450–500 µm or in the middle of the cortical slices (at a depth of 200 µm) at regular intervals of 5 min. Knowing *n* and *D*, the values of α, λ and *k′* can be obtained from the diffusion curves.

### Measurement of Extracellular K^+^ Concentration

The extracellular potassium concentration ([K^+^]_e_) in the mouse cortex *in vivo* during terminal ischemia/anoxia was measured by double-barreled K^+^-sensitive microelectrodes, as described in detail previously [24]. Briefly, the tip of the K^+^-selective barrel of the microelectrode was filled with the liquid ion-exchanger Corning 477317 (currently available as IE 190 from WPI) and back-filled with 0.5 mM KCl, whereas the reference barrel contained 150 mM NaCl. Electrodes were calibrated in a sequence of solutions containing 0.5, 1, 2, 3, 4, 6, 8, 12, 16, 24, 32, 48, 64, 96 and 128 mM KCl, with a background of either 149.5, 149, 148, 147, 146, 144, 142, 138, 134, 126, 118, 102, 86, 54, or 22 mM NaCl to keep the ionic strength of the solution constant. The data were fitted to the Nikolsky equation to determine the electrode slope and interference. Based on these electrode characteristics, the measured voltage was converted to extracellular concentrations.

### Diffusion-weighted Magnetic Resonance Imaging

Diffusion-weighted imaging measurements were performed using an experimental magnetic resonance spectrometer BIOSPEC 4.7 T system (Bruker, Ettlingen, Germany) equipped with a 200 mT/m gradient system (190 µs rise time) and a homemade head surface coil. We acquired a sequence of T_2_-weighted sagittal images to position coronal slices. Two diffusion-weighted images per slice were acquired using the following parameters: Δ = 30 ms, b-factors = 136 and 1825 s/mm^2^, TE = 46 ms, TR = 1200 ms, field of view 1.92×1.92 cm^2^, matrix size = 256×128, four 0.8 mm thick coronal slices, interslice distance = 1.2 mm. Diffusion-weighted images were measured using the stimulated echo sequence. In DW-MRI measurements, the diffusion gradient direction pointed along the rostrocaudal direction. Three pairs of diffusion-weighted measurements (a series of slices acquired with both low and high diffusion weighting) were performed before the magnesium chloride injection. Thereafter, one pair of measurements was done at 5-min intervals for the next 45 min. Maps of ADC_W_ were calculated using the linear least-squares method and analyzed using ImageJ software (W. Rasband, NIH, USA). The evaluated regions of interest (ROI) were positioned using a mouse brain atlas [25] and T_2_-weighted images in both the left and right hemispheres. The minimal area of an individual ROI was 1.2 mm^2^. In each animal, we analyzed four coronal slices from the interval between 0.8 mm frontal to bregma and 3.6 mm caudal to bregma. The resulting eight values of ADC_W_ (two ROIs per slice, four slices) were averaged to obtain a single representative value for comparison with other mice. The resulting ADC_W_ maps were evaluated in the primary somatosensory cortical region, the area corresponding to the site of the TMA measurements.

The reproducibility of ADC_W_ measurements was verified by means of six diffusion phantoms placed on the top of the animals’ heads. The phantoms were made from glass tubes (inner diameter = 2.3 mm, glass type: KS80, Rückl Glass, Otvovice, Czech Republic) filled with pure (99%) substances having different diffusion coefficients. The substances were: 1-octanol, ntridecane (Sigma-Aldrich), isoamyl alcohol, isopropyl alcohol, n-butanol and tert-butanol (Penta, Prague, Czech Republic). The temperature of the phantoms was maintained at a constant 37°C.

### Statistical Analysis

The results of the experiments are expressed as mean plus/minus S.E.M. Statistical analysis of the differences within and between groups was performed using one-way ANOVA with the Tuckey post-test (Instat; GraphPad Software, San Diego, CA, USA). Values of p*<*0.05 were considered significant. Repeated measures within a group of subjects were analyzed using repeated measures ANOVA (Statistica; Statsoft, Tulsa, OK, USA).

## Results

### Immunohistochemical Analysis of Astrocytes

**Figure 2:**
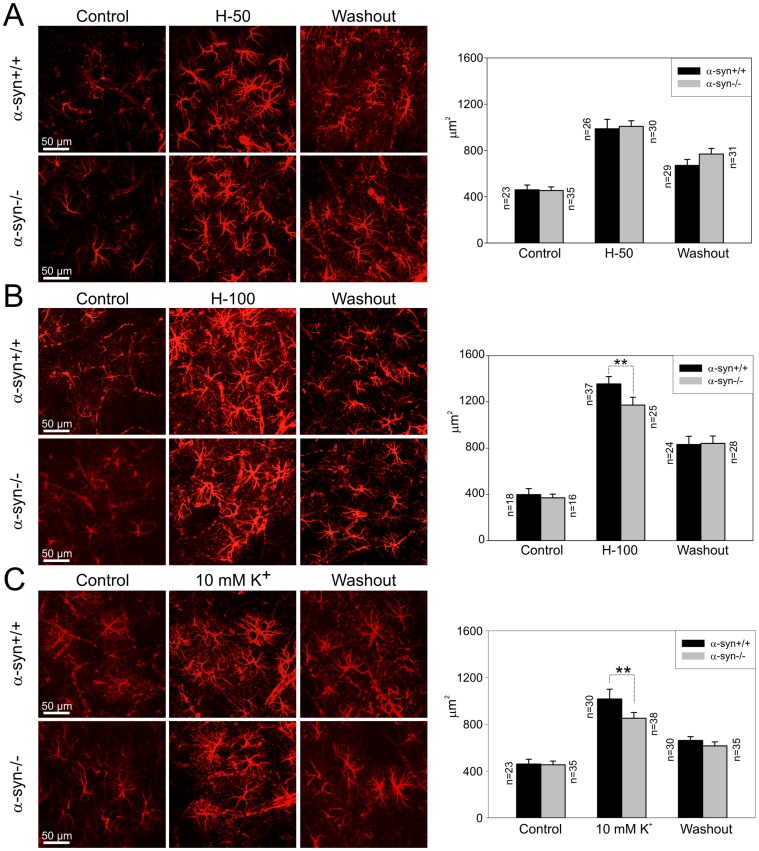
Changes in astrocyte morphology evoked by hypotonic stress and elevated extracellular K^+^. Changes in astrocyte morphology, based on changes in glial fibrillary acidic protein immunoreactivity, were determined in the cortex (layers III–IV) of α-syn +/+ and α-syn −/− mice: 1) prior to (control), 2) after a 30 minute application of hypotonic solutions (H-50 and H-100) or 10 mM K^+^ and 3) following a 60 minute washout. The bar graphs on the right side indicate cell volume changes expressed as changes in the area corresponding to GFAP immunoreactivity; n represents number of cells.

Morphological differences between α-syn +/+ and α-syn −/− cortical astrocytes were analysed prior to, after a 30 min incubation in H-50, H-100 or 10 mM K^+^, and after a 60 min washout. Immunohistochemical staining for GFAP showed no distinct differences in astrocytes in either group prior to incubation (Fig. 2).

The impact of α-syntrophin deficiency on astrocytic swelling was estimated by quantifying GFAP immunoreactivity in individual astrocytes from α-syn +/+ and α-syn −/− mice (Fig. S1). We found that the swelling of α-syn −/− and α-syn +/+ astrocytes was comparable when exposed to hypotonic solution (H-50) (Fig. 2A); however, the swelling of α-syn −/− astrocytes was smaller during their exposure to a more severe hypotonic solution (H-100) or 10 mM K^+^ (Fig. 2B and 2C, respectively). In each model of cell swelling, the analysis showed only a partial recovery of astrocytic volume during washout.

### Diffusion Parameters of the ECS and ADC_W_ in α-syn +/+ and α-syn −/− Mice

**Figure 3:**
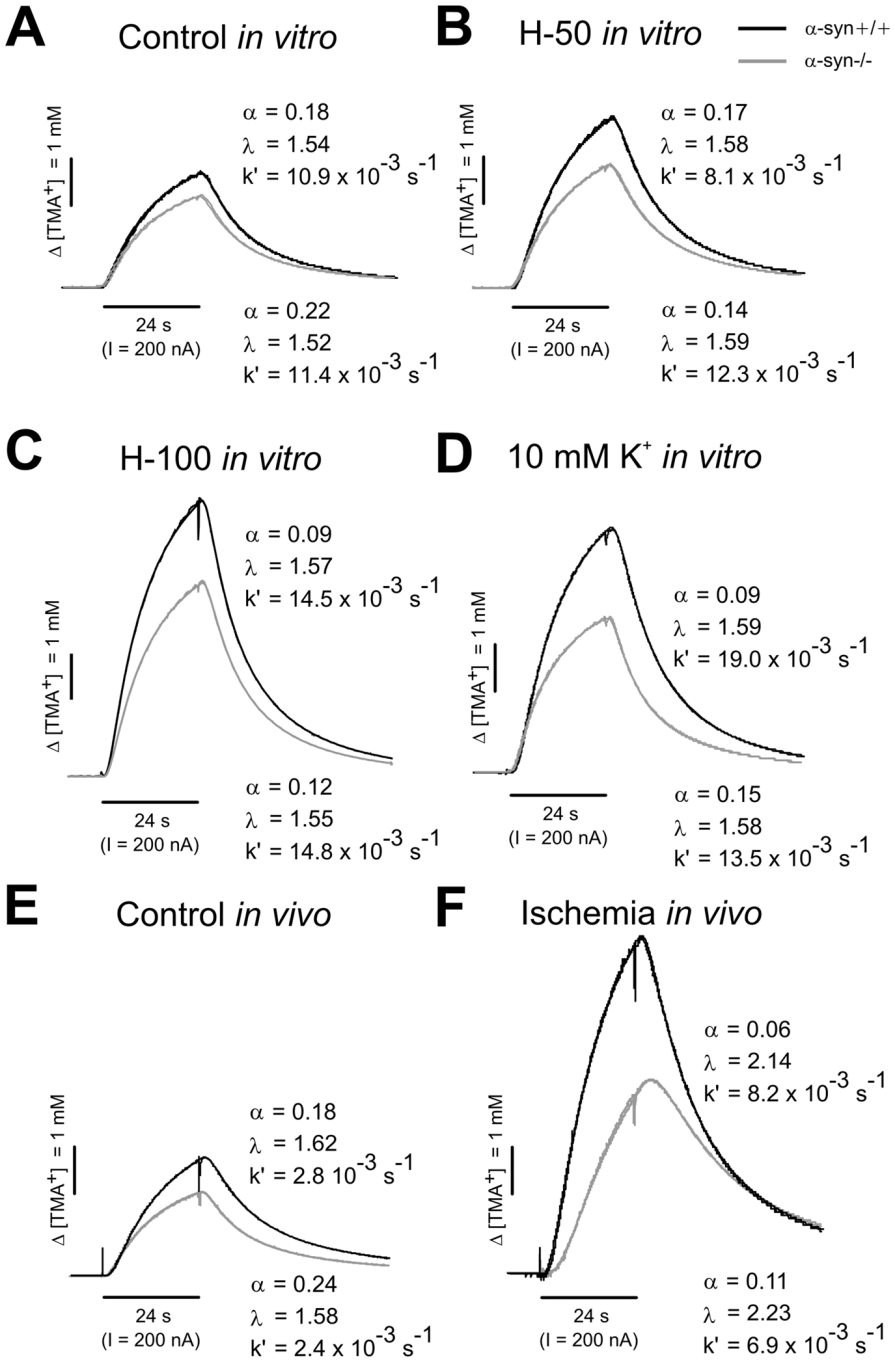
Representative TMA^+^-diffusion curves under resting conditions and during cell swelling. TMA^+^-diffusion curves with the corresponding values of the extracellular volume fraction (α), tortuosity (λ) and non-specific uptake (*k′*) obtained in the cortex of α-syn +/+ and α-syn −/− mice under resting conditions (**A, E**) as well as during acute cell swelling evoked by the application of hypotonic solutions H-50 (**B**), H-100 (**C**) or increased potassium (**D**) *in vitro* or by ischemia/anoxia *in vivo* (**F**). The values of the ECS diffusion parameters were determined by a non-linear curve fitting algorithm operating on the diffusion curve. The amplitude of the curves is inversely proportional to the ECS volume fraction while the shape of the curves reflects tortuosity.

The control values of the ECS volume fraction α measured on coronal slices *in vitro* were significantly higher in α-syn −/− mice compared with α-syn +/+ animals, while the tortuosity λ or non-specific uptake *k′* values did not significantly differ between α-syn +/+ and α-syn −/− mice (Fig. 3A; Table 1). Measurements carried out in the cortex *in vivo* confirmed the significantly higher control values of α in α-syn −/− in comparison with α-syn +/+ mice with no significant difference in λ or *k′* (Fig. 3E; Table 2). Significantly higher control values of ADC_W_ were detected in α-syn −/− mice using DW-MRI.

**Table 1:** Effect of hypotonic stress and elevated K^+^ on the ECS diffusion parameters in α-syn +/+ and α-syn −/− mice *in vitro*.

	α-syn +/+				α-syn −/−			
	α	λ	*k′*×10^−3^s^−1^	n/N	α	λ	*k′*×10^−3^s^−1^	n/N
control values	0.190±0.002	1.502±0.011	9.504±0.700	26/17	0.212±0.003**	1.499±0.008	11.716±1.220	21/13
H-50	0.132±0.004^###^	1.565±0.023^#^	13.990±1.400	9/6	0.157±0.008**^###^	1.554±0.018^#^	13.664±1.679	7/4
wash-out	0.198±0.003	1.542±0.019	9.331±1.301		0.220±0.007*	1.549±0.021	9.735±1.251	
H-100	0.073±0.016^###^	1.589±0.025^#^	23.819±3.335^###^	7/4	0.134±0.015***^###^	1.554±0.025	20.923±1.652^##^	10/5
wash-out	0.222±0.015^#^	1.460±0.022^#^	8.878±2.196		0.283±0.010***^###^	1.512±0.028	14.000±3.044	
10 mM K^+^	0.105±0.014^###^	1.588±0.022^###^	20.579±2.914^###^	10/7	0.154±0.003*^###^	1.569±0.013^#^	15.462±2.884	7/4
wash-out	0.199±0.017	1.566±0.017^#^	9.260±0.797^#^		0.205±0.007	1.515±0.017	13.003±3.763	

The values are presented as mean ± S.E.M. Asterisks (*-p<0.05; **-p<0.01;***-p<0.001) indicate significant differences between the values in α-syn +/+ and α-syn −/− animals; crosshatches (#-p<0.05; ##-p<0.01; ###-p<0.001) indicate significant differences between control values and those obtained under experimental conditions in the same group of animals. The control values of the ECS diffusion parameters from each individual experiment were calculated as the average values extracted from three diffusion curves before application. The mean control value presented in the table is the average of the control values from all in vitro experiments. The mean values of the maximum change during application correspond to the time-point of the maximum decrease of α (i.e., the 25^th^ min in H-50 and H-100 and the 30^th^ min in 10 mM K^+^); the values for washout correspond to the data point at the 90^th^ min. Abbreviations: extracellular space volume fraction (α), tortuosity (λ), non-specific uptake (k*′*), number of animals (N), number of slices (n), hypotonic solutions (H-50 and H-100).

**Table 2:** Effect of terminal ischemia/anoxia on the ECS diffusion parameters, ADC_W_ and extracellular K^+^ concentration in α-syn +/+ and α-syn −/− mice *in vivo*.

	α-syn +/+				α-syn −/−			
	control values	N	terminal ischemia/anoxia	N	control values	N	terminal ischemia/anoxia	N
**α**	0.204±0.003^†††^	14	0.101±0.006^###^	6	0.227±0.003**^††^	11	0.143±0.008***^###^	7
**λ**	1.596±0.010^†††^	14	2.073±0.057^###^	6	1.598±0.010^†††^	11	2.245±0.045*^###^	7
***k*** *′* **x10^−3 ^s^−1^**	3.622±2.421	14	2.102±2.651	6	2.623±2.480	11	5.410±4.270	7
**[K^+^]_e_ (mM)**	2.858±0.218^†^	6	52.370±2.644^###^	6	2.859±0.256^††^	5	53.750±2.635^###^	5
**ADC_W_ (µm^2^s^−1^)**	590±6	6	374±8^###^	6	627±6*	6	413±11*^###^	6

The values are presented as mean ± S.E.M. Asterisks (*-p<0.05; **-p<0.01; ***-p<0.001) indicate significant differences between the values in α-syn +/+ and α-syn −/− animals; crosshatches (#-p<0.05; ##-p<0.01; ###-p<0.001) indicate significant differences between control values and those obtained under experimental conditions in the same group of animals and crosses (^†^-p<0.05; ^††^-p<0.01; ^†††^-p<0.001) indicate significant difference between values in vitro and in vivo. The mean values of the maximum change during terminal ischemia/anoxia correspond to the 30^th^ minute after cardiac arrest. Abbreviations: extracellular space volume fraction (α), tortuosity (λ), non-specific uptake (k*′*), extracellular potassium concentration ([K^+^]_e_), apparent diffusion coefficient (ADC_W_) number of animals (N).

Significant differences in the control values of all three ECS diffusion parameters were found between *in vivo* and *in vitro* conditions in both α-syn +/+ and α-syn −/− mice: α and *k′* values were higher *in vivo*, while λ was higher *in vitro* (Table 1 and 2).

### Effects of Hypotonic Stress *in vitro*

**Figure 4:**
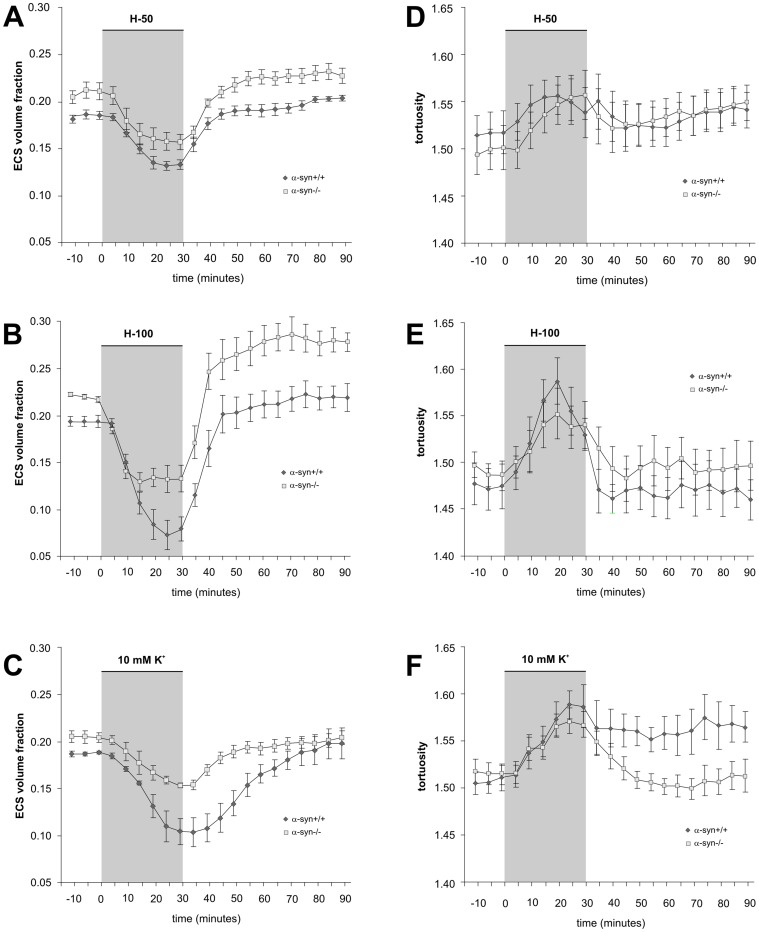
The effect of hypotonic solutions or 10 mM K^+^ on ECS diffusion parameters *in vitro*. The absolute values of the extracellular volume fraction (α) and tortuosity (λ) were measured in cortical slices from the somatosensory cortex of α-syn +/+ and α-syn −/− mice during a 30 min application of hypotonic solution H-50 (−50 mOsmol/l) (**A, B**) and H-100 (−100 mOsmol/l) (**C, D**) or 10 mM K^+^ (**E, F**) and during washout. Each data point represents mean ± S.E.M.

A 30 min superfusion of brain slices with mild hypotonic solution (H-50) led to a significant decrease in α and no increase in λ in α-syn +/+ and α-syn −/− mice (Fig. 4A, B; Table 1). The maximum decrease of the ECS volume reached during the application was larger in α-syn +/+ animals than in α-syn −/− mice (Fig. 3B, Fig. 4A; Table 1). Since there was a significant difference in the control values of the ECS volume between α-syn +/+ and α-syn −/− mice, we set the control values of all experiments to 100% in order to determine the relative changes in ECS volume fraction during perfusion. This analysis revealed that the relative decrease in extracellular volume in α-syn +/+ and α-syn −/− mice during the application of a mild hypotonic stress (of 30.53% ±2.16% and 25.94% ±3.80%, respectively) was not significantly different. In contrast, the application of a more severe hypotonic solution (H-100) evoked a significantly smaller decrease in α in α-syn −/− mice than in α-syn +/+ in both absolute and relative values (Fig. 3C, 4C; Table 1; a relative decrease of 36.79% ±6.05% and 61.58% ±7.53%, respectively). During washout, the values of α overshot the control values in α-syn −/− mice but not in α-syn +/+ animals (Fig. 4C; Table 1). The increase in λ or *k′* evoked by mild or severe hypotonic stress was similar in both animal groups (Fig. 3B, C, 4B, D; Table 1) and fully recovered to control values during washout.

### Effects of Elevated K^+^ *in vitro*

The decrease in α observed during a 30 min superfusion with 10 mM K^+^ was significantly smaller in α-syn −/− mice than in α-syn +/+ animals in both absolute as well as relative values (Fig. 3D, 4E; Table 1; a relative decrease of 27.36% ±1.21% and 44.74% ±5.38%, respectively). The increase in λ evoked by 10 mM K^+^ was similar in both animal groups (Fig. 3D; Fig. 4F; Table 1); however, in contrast to α-syn −/− mice, tortuosity remained elevated even after washout in the α-syn +/+ animals (Fig. 4F; Table 1), indicating the creation of additional diffusion barriers, presumably due to structural changes of the fine astrocytic processes. The increase in *k′* during application was similar in both animal groups (Table 1) and fully recovered to control values during washout.

### Effect of Cardiac Arrest *in vivo*

**Figure 5:**
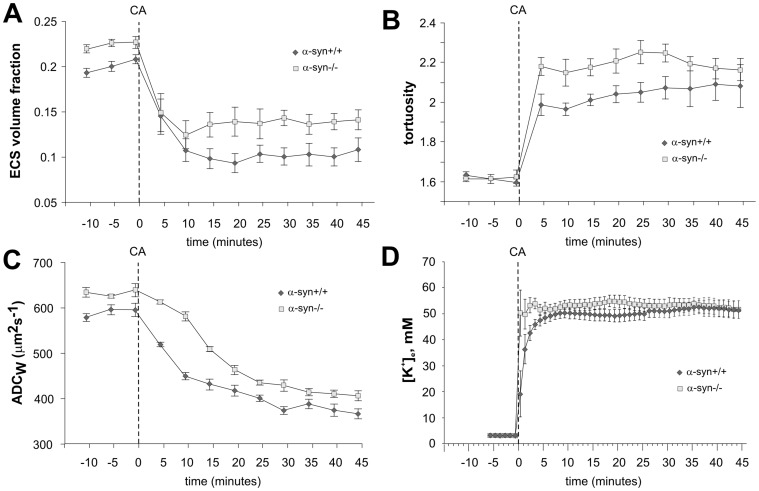
Changes in the ECS diffusion parameters, ADC_W_ and [K^+^]_e_ evoked by terminal ischemia/anoxia *in vivo*. Each data point represents mean ± S.E.M. The control values as well as the final values of the ECS volume fraction α (**A**) and ADC_W_ (**C**) reached during terminal ischemia/anoxia evoked by cardiac arrest (CA) were significantly smaller in α-syn +/+ mice than in α-syn −/− animals and the time course of ADC_W_ changes was slower in the α-syn −/− mice. There was no significant difference in the control values of λ between α-syn +/+ and α-syn −/− mice (**B**). After the onset of terminal ischemia/anoxia, tortuosity (**B**) as well as [K^+^]_e_ (**D**) were significantly higher in α-syn −/− mice compared to α-syn +/+ animals, but the final values did not differ**.**

**Figure 6:**
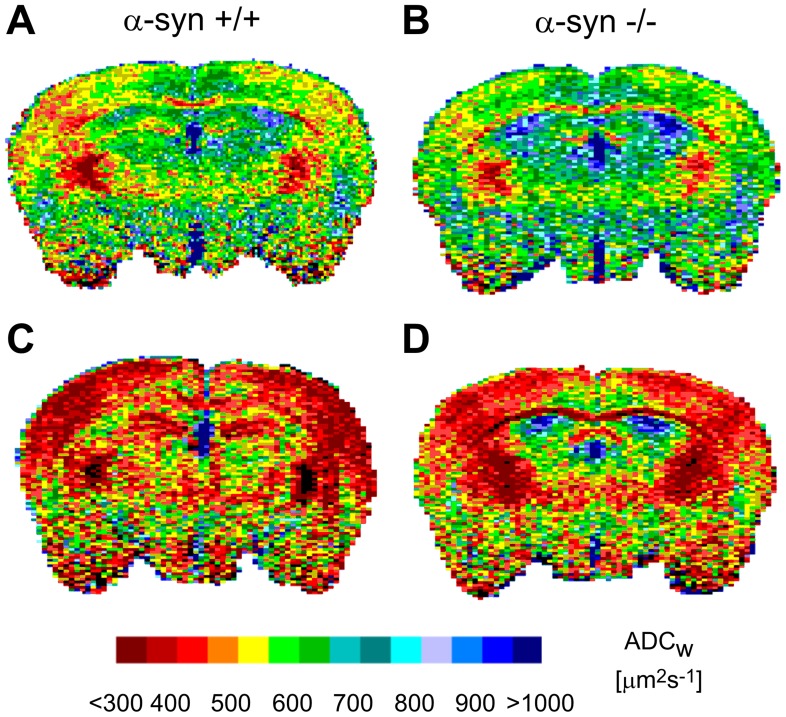
Typical ADC_W_ maps of α-syn +/+ and α-syn −/− mice. ADC_W_ values were averaged in the outlined areas (primary somatosensory cortex). **A** and **B:** The images show ADC_W_ maps of α-syn +/+ and α-syn −/− mice untreated/control mice. **C** and **D:** The images show ADC_W_ maps of α-syn +/+ and α-syn −/− mice 30 minutes after the onset of ischemia. The scale at the bottom of the figure shows the relation between the intervals of ADC_W_ values and the colors used for visualization. Note the smaller ADC_W_ decrease in the α-syn −/− mice mouse when compared to the α-syn +/+ control.

The minimal values of the ECS volume (α) reached after cardiac arrest due to the administration of MgCl_2_ were larger in α-syn −/− mice compared to α-syn +/+ mice, suggesting diminished cell swelling in the α-syn −/− animals (Fig. 5A; Table 2). The maximum decrease of ADC_W_ was also smaller in α-syn −/− mice (Fig. 5C, Fig. 6; Table 2); in addition, the time course of changes in α-syn −/− animals was slowed down in comparison with α-syn +/+ animals (Fig. 5C).

Shortly after the administration of MgCl_2_, the values of λ in α-syn −/− mice reached higher levels than in α-syn +/+ animals (Fig. 5B), reflecting a more rapid increase in the number of obstacles for diffusion in the ECS of the α-syn −/− mice. However, the final values of λ 35–45 min after the induction of cardiac arrest were not significantly different between the two types of animals (Fig. 5B; Table 2).

The increase of [K^+^]_e_ in α-syn +/+ and α-syn −/− mice immediately after terminal cardiac arrest displayed a steep rise, which was faster and reached its maximum in α-syn −/− animals in about 4 min after cardiac arrest. In α-syn +/+ animals, [K^+^]_e_ increased more slowly and reached maximum values 9 min after MgCl_2_ administration (Fig. 5D). There were no significant differences between these two animal groups in the final values of [K^+^]_e_ (Fig. 5D; Table 2).

## Discussion

Using the real-time iontophoretic method both *in vivo* and *in vitro*, we have demonstrated that the ECS volume fraction under resting conditions is higher in mice lacking the α-syn protein, while there was no difference in tortuosity between α-syn +/+ and α-syn −/− mice, which is in agreement with another TMA study performed on AQP4-deficient mice *in vivo* [26]. Studies using electron microscopy have revealed that perivascular and subpial astroglial end-feet are swollen in the brains of α-syn −/− mice in the basal state, compared to α-syn +/+ animals, indicating the compensatory shrinkage of the surrounding ECS [27]. Thus, it was suggested that α-syn −/− mice have a reduced clearance of the water generated by brain metabolism and that AQP4 channels facilitate the release of water from astrocytes into the brain capillaries under resting conditions. It may seem controversial that our study shows a higher ECS volume fraction in α-syn −/− mice, but the RTI-TMA method describes the average properties of the ECS over a range of about 0.5 mm^3^, including the extracellular volume around the somata and processes. Interestingly, Zeynalov and colleagues [28] have found reduced brain water content in α-syn −/− animals with no differences in the baseline level of serum osmolality. However, a recent study by Haj-Yasein and co-authors [29] in glial-conditional AQP4-deficient animals showed increased brain water content and delayed postnatal water resorption, which might be associated with an increased ECS volume.

There are controversial reports in the literature about the tortuosity changes in AQP4-deficient mice observed using different methods. While the study of Yao and colleagues [26] is in agreement with our findings, diffusion studies with dextran polymers using fluorescence recovery after photobleaching (FRAP) showed, in contrast, a decrease of about 10–20% in tortuosity in the neocortex of AQP4−/− mice [30], [31]. Since the recording intervals are significantly shorter and the molecular weight of the used extracellular marker is higher in the FRAP method compared to RTI-TMA method, the diffusion parameters measured by these methods may differ. Moreover, a decrease in the tortuosity of dextran may only reflect the increased volume that is available for its diffusion, which the FRAP method cannot detect. Interestingly, a study employing the integrative optical imaging (IOI) method to quantify the diffusion of dextran polymers also reported no difference in tortuosity in the neocortex of AQP4 knock-out and wild type mice [32]. Further analysis showed that the FRAP method detects only locally restricted diffusion, while the IOI method examines diffusion parameters from the average volume [32]. Currently, the RTI-TMA method is the only method allowing for the determination of the absolute values of ECS volume fraction and tortuosity, as well as their dynamic changes both *in vivo* and *in vitro* [1], [21]. Total diffusibility of the tissue was determined in this study by DW-MRI, showing that the decrease in ADC_W_ was milder in α-syn−/− than in α-syn +/+ mice, similarly as the decrease in α. Moreover, the time course of ADC_W_ changes, but not of α changes, was slower in the α-syn −/− animals. Correlating the RTI and DW-MRI methods is not simple. While the RTI method measures the geometrical and viscosity parameters of the extracellular space, DW-MRI measures the overall diffusion coefficient in the tissue. Changes in ADC_W_ usually correlate well with changes in the ECS parameters during physiological states as well as in various pathologies [33] because geometrical changes in the ECS are related to water movement between compartments with different diffusion coefficients (i.e., the extracellular and intracellular space). On the other hand, ADC_W_ also reflects diffusion in the intracellular space and so-called dead spaces that cannot be reached by diffusing TMA^+^ molecules. Moreover, since ADC_W_ reflects both α and λ changes, the correlation between ADC_W_ and the ECS diffusion parameters is valid only for acute cell swelling such as ischemia/anoxia and not for chronic states with the formation of additional diffusion barriers [33]. The ECS diffusion parameters provide important information for experimental and clinical neuroscience research, however, the methods employed for the direct measurement of these parameters, such as RTI, FRAP or IOI, are less suitable for clinical use because of their invasiveness. On the other hand, the non-invasiveness, good spatial resolution and widespread availability of suitable scanners have made DW-MRI an important tool for studying diffusion parameters in the brain in clinical research. Although DW-MRI does not provide complete information about the diffusion parameters in the ECS, changes in ADC_W_ can be better interpreted when correlated with the ECS diffusion parameters measured by the RTI method.

Interestingly, our measurements in coronal slices revealed higher control values of α and *k′* but smaller control values of λ than were found under *in vivo* conditions. Small differences between the ECS diffusion parameters measured *in vitro* and *in vivo* have been reported previously (for review see [21]) and may result from increased water accumulation in the tissue (increased α and *k′*) and from cut cell processes and/or washing out of the extracellular matrix (decreased λ) in slices.

It has been suggested that α-syn protein regulates AQP4 localization in the astrocytic end-feet [10] since, as found in α-syn −/− mice, most of the AQP4 channels disappeared from the astrocytic end-feet that face blood vessels, although the total number of brain AQP4 channels did not change. The redistribution of AQP4 channels without any decrease in their total number may explain the fact that we did not detect any significant difference in the relative ECS volume decrease between α-syn +/+ and α-syn −/− mice during mild hypotonic stress. However, since α-syn −/− mice have a larger ECS volume fraction under control conditions, the absolute values of α during hypotonic stress do not decrease as much as in α-syn +/+ mice, meaning that neuroactive substances might reach a neurotoxic concentration more slowly. The differences between α-syn +/+ and α-syn −/− mice in relative decrease of α were revealed only by the more severe hypotonicity of the perfusing solution (H-100), which might indicate that water transport through aquaporin channels becomes more important during pathological conditions then during physiological states. This hypothesis is supported by the fact that an increase in non-specific uptake during severe hypotonic stress, increased potassium concentration or ischemic conditions, which reflects changes in the permeability of the cell membranes and/or capillaries and might be an additional factor affecting transmembrane ion and water movement and thus cell swelling, did not differ between α-syn +/+ and α-syn −/− mice.

The application of 10 mM K^+^ evoked more pronounced changes in ECS volume than did mild hypotonic stress and highlighted the differences between α-syn +/+ and α-syn −/− mice. This might be due to the more complex mechanisms occurring in the tissue during [K^+^]_e_ elevation, which involve KCl uptake through Na^+^-K^+^-2Cl^−^ co-transporters that creates a driving force for the influx of water [34] and the release of excitatory amino acids (EAA) from astrocytes, mediated by volume-sensitive organic anion channels [35] or the different proportion of swelling between the cell soma and the processes. Quantifying the changes in GFAP immunoreactivity disclosed that the knockout of α-syn alters astrocyte swelling when exposed to H-100 hypoosmotic solution or 10 mM K^+^. The finding, that the application of H-50 hypotonic solution was not sufficient to reveal such alterations in astrocyte swelling, further supports the above-mentioned hypothesis. Nevertheless, we should consider that quantification of GFAP staining only gives an approximate estimation of astrocytic volume changes in general, because these intermediate filaments do not fill the entire cell volume. Thus, the supposed control cell volume in our morphometry analysis may be underestimated and the real relative change during cell swelling might be smaller. From our study, it is evident that changes in astrocytic volume estimated from GFAP immunoreactivity and changes in ECS volume are not in direct relationship. While GFAP analysis in our study showed only a partial recovery of astrocytic volume after experimentally evoked cell swelling, the RTI method detected a full recovery or even an overshoot in the values of the ECS volume fraction α. Since the ECS volume changes reflect reciprocally the volume changes in all cellular elements of the tissue, including neurons, oligodendrocytes, polydendrocytes, microglia, pericytes, and endothelial cells, we can hypothesize that intense volume regulation of some of these cell types may compensate for the insufficient recovery of astrocytes. Moreover, it is important to realize that staining for GFAP is rather limited to the primary and secondary processes. Thus, a rebuilding of the tertiary or quaternary processes, which is, as we believe, associated with the formation of additional diffusion barriers and a persistent increase in tortuosity after increased K^+^ application, cannot be detected. A more detailed evaluation of the possible fine structural changes in the finest astrocytic processes is required, which would bring additional information about astrocyte swelling/volume recovery in the cortex of α-syn −/− mice, e.g., using confocal morphometry to analyze astrocytes expressing enhanced green fluorescent protein under the control of the human GFAP promoter [36], [37].

While 10 mM K^+^ and mild hypotonic stress (H-50) are models of physiological cell swelling (for example, as the result of intensive neuronal activity), global or terminal ischemia/anoxia represents a severe pathological condition with massive cell swelling and a dramatic increase in extracellular potassium [38], leading to the activation of many volume regulatory mechanisms, such as the increased release of glutamate, aspartate, chloride and taurine, which help reduce the intracellular water volume [34]. Our *in vivo* data show that the onset of the steep rise in [K^+^]_e_ directly after terminal ischemia/anoxia induction is faster in α-syn −/− mice, compared to α-syn +/+ animals. This fast, steep rise of [K^+^]_e_ in α-syn −/− mice may reflect the inability of astrocytes to distribute K^+^ in the extracellular space by spatial buffering as well as across the blood-brain barrier. Altered K^+^ clearance, manifested mostly by a prolonged decay time to the baseline values after a stimulation-evoked [K^+^]_e_ increase, was also confirmed in other studies in α-syn or AQP4 knock-out mice [9], [11], [12]. Biochemical data indicating that AQP4 and Kir4.1 proteins might be part of the same complex [39] as well as studies using electron microscopy and co-immunoprecipitation analyses showing the subcellular co-localization of AQP4 and Kir4.1 in the retina [40], [41] gave rise to the hypothesis that AQP4 channels have a functional relationship with inwardly rectifying K^+^ channel subunit 4.1 (Kir4.1), which is involved in spatial buffering [42]. However, more recent studies did not support this idea, suggesting that some other mechanisms explaining the slower K^+^ reuptake in AQP4−/− tissue, e.g. reduced glial cell swelling and a larger extracellular space, might be involved [12], [43]. Strohschein and colleagues [12] further showed facilitated spatial buffering associated with enhanced glial coupling in AQP4−/− mice. Moreover, a recent study performed using glia-specific conditional Kir4.1 knock-out mice (cKir4.1−/−) showed altered spatial buffering of K^+^ with an unchanged distribution of AQP4 channels in these animal [44], indicating that the exact relationship between Kir4.1 and AQP4 has not yet been fully clarified.

Our *in vivo* results show that 30 min after the induction of terminal ischemia/anoxia, the ECS volume fraction was larger in α-syn −/− mice, indicating less cell swelling. These results correspond to our *in vitro* measurements and to recent studies showing that in α-syn −/− mice, brain edema is reduced and attenuated during transient cerebral ischemia [27]. Despite the larger ECS volume fraction in α-syn −/− animals, the tortuosity values during the early phase (up to 35 min) of terminal ischemia/anoxia were higher in α-syn −/− mice compared to α-syn +/+ animals, while the final values of tortuosity did not significantly differ. This suggests the appearance of additional diffusion barriers in the ECS due to transient structural changes probably associated with the initially higher extracellular K^+^ concentration under ischemic conditions and altered spatial buffering in these mice. Indeed, our current data as well as our previous studies show that increased K^+^ induces morphological changes, especially in fine astrocytic processes, that are associated with a large increase in tortuosity [45]. Moreover, we have also shown that pre-exposure to high K^+^ concentrations, an early event leading to astrogliosis, causes not only morphological changes in astrocytes, but also changes in their membrane properties and cell volume regulation [46]. The smaller degree of astrocyte swelling after high potassium pre-exposure may thus contribute to the smaller decrease in ECS volume found in the α-syn −/− cortex after the induction of terminal ischemia/anoxia.

Our data indicate that the removal of the perivascular pool of AQP4 due to α-syntrophin deletion reduces edema formation, especially under pathological conditions and during states associated with elevated K^+^, which may be related to altered K^+^ transport in these animals. A larger initial extracellular volume could also serve as a protective factor that buffers any increase in the concentration of potentially neurotoxic substances and slows down the process of cell swelling. On the other hand, impaired water movement across cell membranes may delay the recovery and normalization of the volume conditions.

This is the first study that reveals the functional involvement of α-syntrophin protein in the changes in both ECS diffusion parameters during cell swelling evoked by physiological as well as pathological stimuli. Changes in tortuosity, reflecting the number and extent of diffusion barriers, and in the extracellular volume fraction affect the movement of neuroactive substances as well as trophic factors and thus may modulate the extent of the damaged area, the process of healing and/or drug distribution. Understanding the detailed mechanisms underlying the movement of water and ions (especially K^+^) across the cell membrane could reveal new targets for potential therapeutic intervention during serious human pathologies associated with cell swelling, such as stroke or blood-brain barrier damage.

## Supporting Information

Figure S1 Quantification of GFAP staining in individual astrocytes. **Left:** A superimposed image of GFAP staining in cortical astrocytes obtained by overlaying 20 individual confocal planes. **Right:** The superimposed image has been digitally filtered, and a red area (marked by yellow) was used for the quantification of GFAP staining. The area corresponding to the GFAP immunoreactivity of each astrocyte was calculated in clearly defined regions of interest (ROS). Here we show examples of such ROS that correspond to GFAP staining in individual astrocytes; individual ROS are highlighted by blue, green, pink and white colors.
(TIF)

Methods S1 (DOCX)
