# Supplementary figures and images for: The Impact of Alpha-Syntrophin Deletion on the Changes in Tissue Structure and Extracellular Diffusion Associated with Cell Swelling under Physiological and Pathological Conditions
Source: PLoS One. 2013 Jul 5;8(7):e68044. doi: 10.1371/journal.pone.0068044 (PMC3702576; doi:10.1371/journal.pone.0068044)

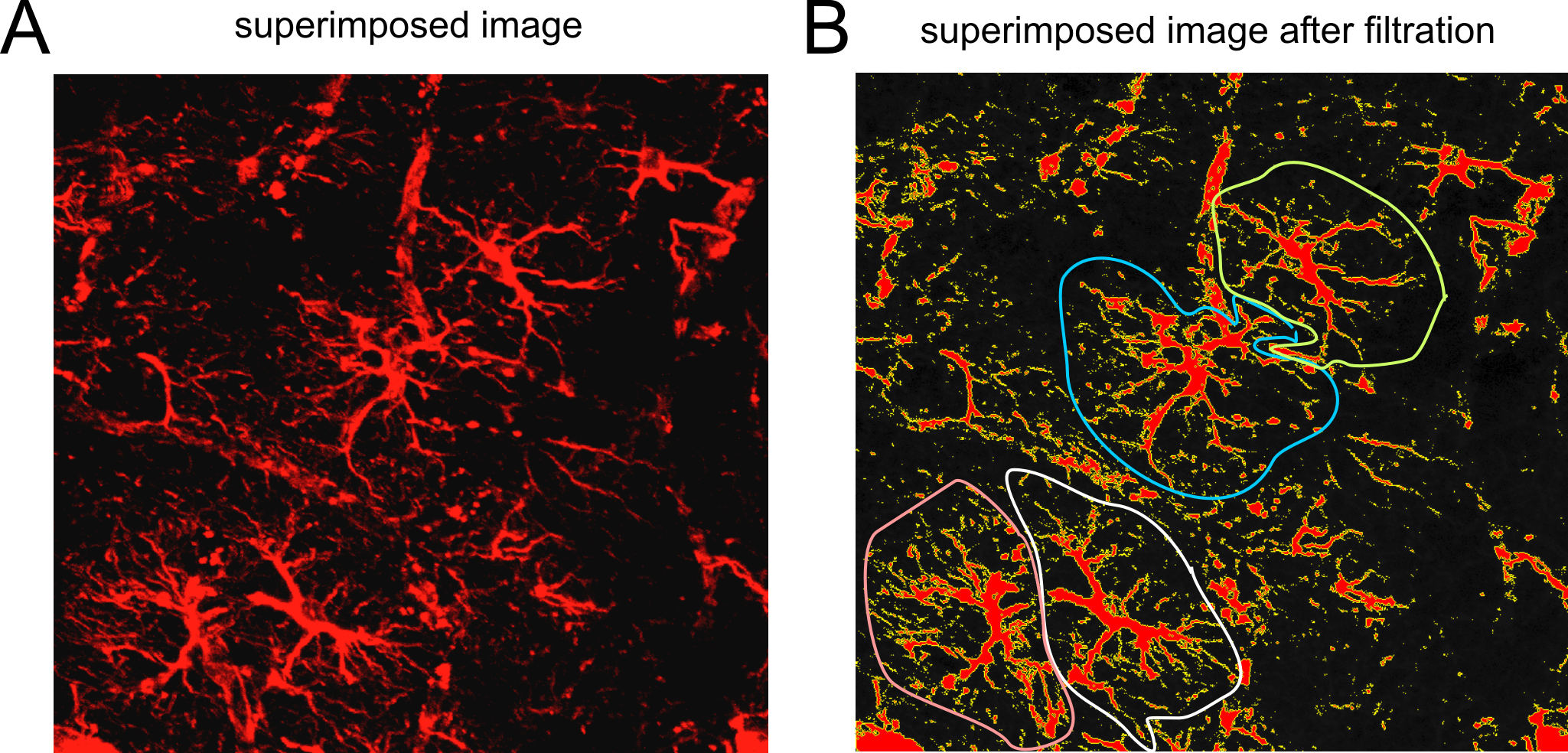

Supplement: Figure S1 — Quantification of GFAP staining in individual astrocytes. Left: A superimposed image of GFAP staining in cortical astrocytes obtained by overlaying 20 individual confocal planes. Right: The superimposed image has been digitally filtered, and a red area (marked by yellow) was used for the quantification of GFAP staining. The area corresponding to the GFAP immunoreactivity of each astrocyte was calculated in clearly defined regions of interest (ROS). Here we show examples of such ROS that correspond to GFAP staining in individual astrocytes; individual ROS are highlighted by blue, green, pink and white colors. (TIF) [file pone.0068044.s001.tif]
