# Supplementary material for: The Impact of Alpha-Syntrophin Deletion on the Changes in Tissue Structure and Extracellular Diffusion Associated with Cell Swelling under Physiological and Pathological Conditions
Source: PLoS One. 2013 Jul 5;8(7):e68044. doi: 10.1371/journal.pone.0068044 (PMC3702576; doi:10.1371/journal.pone.0068044)
Supplement: Methods S1 — (DOCX) [file pone.0068044.s002.docx]

Supporting information

**SI Methods**

***Quantification of GFAP staining in individual astrocytes***

Changes in GFAP immunoreactivity in response to hypotonic stress or 10 mM K^+^ were quantified using a previously described method [1]. The GFAP staining was recorded as a set of two-dimensional (2D) sectional images with a resolution of 1024x1024 pixels using a Leica TCS SP system confocal microscope (Leica Germany). Alexa 488 was excited by an Ar laser set at 488 nm, and the emitted signal was recorded over the range of 510 – 552 nm using a TD488/543/633 filter. Each image was sectioned into 40 – 50 consecutive 2D images (at a uniform spacing of 0.5 µm), which were superimposed for further analyses. The parameters of the confocal microscope scanning, such as laser intensity, pinhole, gain and offset, were kept constant during the scanning of all samples. Image processing and morphometric measurements were performed using the program CellAnalyst developed at the Department of Cellular Neurophysiology, Institute of Experimental Medicine, Prague, Czech Republic [2]. The area corresponding to GFAP immunoreactivity was estimated in individual cells in cortical slices prior to (control), during and after (washout) the application of hypotonic or 10 mM K^+^ solutions (Figure S1A, B). Three animals were used for each insult (H-50, H-100, 10 mM K^+^). GFAP immunoreactivity was evaluated in 3 slices from each animal: the 1^st^ slice prior to incubation, the 2^nd^ slice during the cell swelling evoking insult, and the 3^rd^ slice during washout. In each slice 3 zones, 250x250 μm, were analyzed; only whole astrocytes were evaluated in each zone (2-4 cells in each zone).

**SI References:**

1. Chvatal A, Anderova M, Kirchhoff F (2007) Three-dimensional confocal morphometry - a new approach for studying dynamic changes in cell morphology in brain slices. J Anat 210:671-683.

2. Benesova J, Rusnakova V, Honsa P, Pivonkova H, Dzamba D, et al. (2012) Distinct expression/function of potassium and chloride channels contributes to the diverse volume regulation in cortical astrocytes of GFAP/EGFP mice. PLoS One. 7:e29725.
